# Assessment and monitoring of crop water use and productivity in response to climate change

**DOI:** 10.1017/S002185961800076X

**Published:** 2018-07

**Authors:** Anna Dalla Marta, Josef Eitzinger, Kurt-Christian Kersebaum, Mladen Todorovic, Filiberto Altobelli

**Affiliations:** 1 Department of Plant, Soil and Environmental Science, University of Florence, piazzale delle cascine 18, Florence 50144, Italy; 2 Department of Water, Atmosphere and Environment (WAU), Institute of Meteorology (BOKU-Met), University of Natural Resources and Applied Life Sciences (BOKU), Peter-Jordan Str. 82, Vienna A-1190, Austria; 3 Landscape Systems Analysis, ZALF, Eberswalder Str. 84, Müncheberg 15374, Germany; 4 Land and Water Management, Centre International de Hautes Etudes Agronomiques Mediterraneennes, Via Ceglie 9, Valenzano, Bari 70010, Italy; 5 Center for Policies and Bioeconomy, CREA - Consiglio per la ricerca in agricoltura e l'analisi dell'economia agrarian, Via Po 14, Rome, Lazio 00195, Italy

**Keywords:** Irrigated agriculture, rainfed agriculture, water management

Climate and agriculture are inherently linked through the agro-ecological zones specifying the best-growing conditions for each crop. The evapotranspirative demand of the atmosphere is a function of the site-specific weather conditions and affects the crop water needs for optimal growth. Moreover, the air temperature determines the duration of the crop growth cycle, which, together with other factors, triggers growth and yield, and causes a chain of successive effects on socio-economic development, food security and trade.

Nowadays, there is strong evidence of climate change impact on agricultural water use and production. The availability of land and water resources for agricultural use is decreasing, while the demand for food is increasing due to population growth and changes in diets. Therefore, there is a powerful need to strengthen the agricultural water sector with new management strategies, techniques and measures that can improve and stabilize crop water productivity under on-going and future climate limitations. Consequently, the sustainable and efficient management of land and water resources, defined through an interdisciplinary approach, high-level scientific information and the development and application of advanced technological solutions is a primary goal.

The use of advanced tools and real-time data such as remote sensing, updated climatic databases, seasonal weather forecasting, climatic projections/scenarios, hydrological, crop-growth and soil–water balance models, as well as their integration, represents a key strategy for the sustainability of both irrigated and rainfed agriculture. Accordingly, the set of manuscripts included in this Themed Issue aims to provide an extensive insight into the most recent key tools and agricultural strategies, focusing on the assessment and improvement of crop water productivity under different climatic conditions and at different scales. As is shown clearly, the use of such instruments allows a detailed analysis of interactions between soil–plant–atmosphere continuum and management practices and, therefore, many new and complex scenarios can be taken into account.

The use of remotely sensed data has become more common to support management, monitoring and controlling activities at different spatial and temporal scales. Papadavid and Toulios give an overview of the use of remote sensing for estimating crop evapotranspiration and yield at regional level, while Thaler *et al*. describe the advantages of satellite-based soil moisture products as key information for the estimation of crop–soil water balance. On the contrary, the operational use of remote sensing for water management in agriculture, and the impact of related information, should also be considered. This aspect is described in two papers by Altobelli *et al*. that analyse the use of satellite-based irrigation advisory services, as well as the use of the water footprint as an indicator of sustainability, from a socio-economic perspective.

The use of crop and cropping system models, and their forcing with numerical weather predictions (NWPs), allows investigation of the performance of crops under different climates and management techniques, providing key evidence about the best adaptation strategies that should be adopted to minimize the impact of climate change on agricultural production. In this respect, Pohankova *et al*. analyse the use of crop growth models for assessing crop water productivity of barley. Furthermore, the integration of crop models with NWPs is thoroughly investigated by Lalic *et al*. for winter wheat and summer crops, by Stricevic *et al*. for maize grown under present and future climate of Bosnia and Herzegovina, and by Ventrella *et al*. for tomato in Southern Italy. Further, the combination of NWPs and VIS–NIR satellite images is presented by Chirico *et al*. as a strategy for long-term sustainability of irrigated agriculture in Southern Italy.

Finally, the results of field experiments are used as the backbone to develop and improve modelling approaches at different scales and for different purposes. In this context, a set of measured data, information and scientific breakthrough knowledge about the adoption of alternative practices in agriculture is presented by Deelstra for water productivity improvement of rice cultivated in an Indian case study, and by Balestrini *et al*. using biological and chemical priming agents to improve the crop performances under water stress conditions. Hence, as a whole, this Themed Issue aims to provide evidence that field experiments, crop and agro-ecological system simulation models and observations from remote sensing can be applied advantageously, singularly or combined, in order to improve model parameterization, the knowledge of complex interactions, forecasting capabilities and develop tools suitable to support agronomic and environmental decision-making process under present and future climate change.

